# cGLRs Join Their Cousins of Pattern Recognition Receptor Family to Regulate Immune Homeostasis

**DOI:** 10.3390/ijms25031828

**Published:** 2024-02-02

**Authors:** Vijay Kumar, John H. Stewart

**Affiliations:** Laboratory of Tumor Immunology and Immunotherapy, Department of Surgery, Morehouse School of Medicine, Atlanta, GA 30310, USA; jstewart@msm.edu

**Keywords:** PRRs, cGLRs, cGAS, STING, type 1 IFN, immunity, inflammation

## Abstract

Pattern recognition receptors (PRRs) recognize danger signals such as PAMPs/MAMPs and DAMPs to initiate a protective immune response. TLRs, NLRs, CLRs, and RLRs are well-characterized PRRs of the host immune system. cGLRs have been recently identified as PRRs. In humans, the cGAS/STING signaling pathway is a part of cGLRs. cGAS recognizes cytosolic dsDNA as a PAMP or DAMP to initiate the STING-dependent immune response comprising type 1 IFN release, NF-κB activation, autophagy, and cellular senescence. The present article discusses the emergence of cGLRs as critical PRRs and how they regulate immune responses. We examined the role of cGAS/STING signaling, a well-studied cGLR system, in the activation of the immune system. The following sections discuss the role of cGAS/STING dysregulation in disease and how immune cross-talk with other PRRs maintains immune homeostasis. This understanding will lead to the design of better vaccines and immunotherapeutics for various diseases, including infections, autoimmunity, and cancers.

## 1. Introduction

Pattern recognition receptors (PRRs) play critical roles in embryonic development, immune homeostasis, neurodevelopment, and neurodegeneration. PRRs are highly conserved germline-encoded proteins that recognize microbe/pathogen-associated molecular patterns (MAMPs or PAMPs) and death/damage-associated molecular patterns (DAMPs) [1,2,3]; thus, they regulate innate and adaptive immunity [1,4,5] and contribute to the pathogenesis of many diseases ranging from infections to cancers [5,6,7,8,9,10,11]. Traditionally, PRR family members have included toll-like receptors (TLRs), C-type lectin receptors (CLRs), nucleotide-oligomerization domain (NOD)-like receptors (NLRs), absent in melanoma-2-like receptors (ALRs), retinoic acid-inducible gene (RIG)-1-like receptors (RLRs), C-type lectin receptors (CLRs), and different scavenger receptors (SRs) [2,9,12,13,14,15,16,17]. Cytosolic guanosine monophosphate (GMP)-adenosine monophosphate (AMP) synthase (cGAS)-like receptors (cGLRs) have been recently recognized as new PRRs that potentially interact with other PRRs to regulate immune homeostasis. The current article is focused on cGLRs and their role in the regulation of immune homeostasis via the cGAS/STING signaling pathway and their interactions with other PRRs. The article concludes with the addition of cGLRs in the PRR family and (cGAS/STING signaling) ideas of how to leverage them to revolutionize the field of vaccinology and immunotherapeutics for cancer and infectious diseases via cross-talking with other PRR family members.

## 2. Emergence of cGLRs (Harboring cGAS and STING) as a Novel Family of PRRs

cGLRs have emerged or evolved as critical PRRs providing defense through different mechanisms in prokaryotes and eukaryotes, including humans. Table 1 shows different cGLRs and their known ligands. The first enzyme responsible for synthesizing cyclic guanosine monophosphate (GMP)-adenosine monophosphate (AMP) (cGAMP) or cyclicdinucleotides (CDNs), dinucleotide cyclase (DncV), or cGAS-DncV-like nucleotidyltransferase (CD-NTase), evolved in bacteria and has been reported in *Vibrio cholerae*. CD-NTases generating CDNs, like animal cGASs, are very diverse proteins numbering over 6000 to date [18]. In addition to producing CDNs, bacterial CD-NTases can also produce cyclic trinucleotides (CTNs) and linear oligonucleotides [19,20]. Although DncV lacks primary sequence homology to h-cGAS (synthesizes 2′3′-cGAMP), it synthesizes conventional 3′3′- cGAMP involved in bacterial chemotaxis [21,22]. Bacteria encode thousands of cGAS/DncV-like NTases (CD-NTases) to control their highly divergent anti-phage defense system. Furthermore, DncV NTases, cGAS homologs, and 2′-5′-oligoadenylate synthase 1 (OAS1, having aspartic acid (Asp) in place of E225) have five highly conserved active sites [22]. The CD-NTases of the cyclic oligonucleotide-based antiphage signaling system (CBASS, analogous to the cGAS/STING signaling pathway of metazoans) that are activated by the binding of a folded fragment of RNA share distant homology with the cGAS [23,24,25,26,27]. Furthermore, CBASS-activating bacteriophage RNA (cabRNA), via binding to the surface of positively charged CdnEO3 cyclase (a bacterial cGAS), promotes cGAMP synthesis to activate the CBASS-mediated immune response [28].Thus, the CBASS in the bacteria producing cGAMP serves as a defense system against bacteriophages [29]. The bacterial cGAS or CBASS comprises a four-gene operon that encodes bacterial cGAS, which is associated with phospholipase, two enzymes with the eukaryotic-like domains E1 and E2, and a Janus kinase-binding protein (JAB) domain, which provides resistance against several bacteriophages. cGAMP production activates phospholipases to disintegrate membranes and bacterial cell death before the completion of the bacteriophage life cycle or the production of mature bacteriophage [29].

Phylogenetic studies have indicated that animal cGAS, or chromosome 6 open reading frame 150 (C6orf150), and STING date back to the origin of choanoflagellate (nearest free-living unicellular and colonial flagellates related to metazoans) called *Monosiga brevicollis* [22]. However, cGAS and STING proteins have substantially different origins, as STINGs arose via convergent domain shuffling in bacteria and eukaryotes, while cGAS homologs or eukaryotic CD-NTases arose due to multiple horizontal gene transfer (HGT) events from bacteria to eukaryotes [18]. Notably, cGAS homologs of invertebrates may not always recognize dsDNA as a PAMP/MAMP and DAMP. Thus, within metazoans, homologs of both cGAS and STING are present as early as in cnidarians including *Nematostella vectensis* and *Hydra magnipapillata* [22]. cGAS and STING are present in all Drosophila species, many non-Drosophila arthropods, and almost all chordates except torafugu *Takifugu rubripes*. Interestingly, cGAS and STING are absent in the flatworm *Schistosoma mansoni* and nematodes such as *Caenorhabditis elegans* [22,24]. Furthermore, they are present in *H. magnipapillata* (*Hydra*), *Tribolium*. *Castaneum* (*Red Flour Beetle*), *Drosophila virilis*, *Drosophila persimilis*, *Drosophila pseudoobscura*, cephalochordate *Brachiostoma floridae* (Florida lancelet), and *Danio rerio* (zebrafish), each of which has two cGAS homologs. Also, *H. magnipapillata* contains three STING candidates (STING-1, STING-2, and STING-3), while *B. floridae* harbors two STING candidates (STING-1 and STING-2) (Table 1) [22].

**Figure 1:**
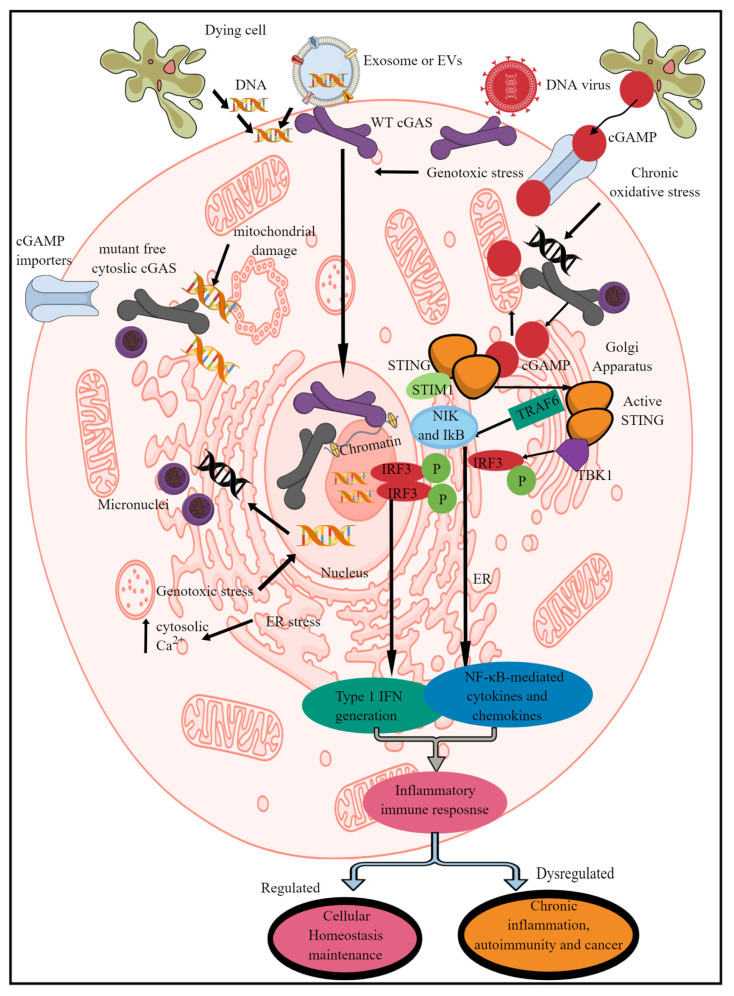
Schematic representation of mammalian cGLR (cGAS/STING signaling) activation and downstream effects. cGAS recognizes cytosolic dsDNA, which can be foreign (pathogen-derived) or self- or adjacent-cell-generated (Mt-DNA and micronuclei generated due to genotoxic or oxidative stress) as a PAMP/DAMP. The enzymatic activity of cGAS cleaves cytosolic dsDNA into CDNs called cGAMP. STING recognizes cGAMP, which induces its transfer to ERGIC and the Golgi apparatus. For example, in the non-activated stage, it remains in the ER via binding to STIM-1 to retain its position in the ER membranes. The STING translocation to the Golgi apparatus phosphorylates (denoted by “P” in figure),TBK1 which induces IRF3 phosphorylation and induction of IRF3-dependent type 1 IFN genes. Active STING also phosphorylates TRAF6, which induces downstream signaling molecules (NIK and IκB) to initiate the transcription of NF-κB-dependent pro-inflammatory genes. Cells such as immune cells also express cGAMP importers, which import cGAMP generated by distant cells to initiate STING signaling without the involvement of cGAS itself. Notably, cGAS located in the nucleus is unable to recognize and bind to nuclear DNA to initiate the pro-inflammatory immune response. Kindly see the text for details.

cGAS/STING/TBK1 axis-dependent type 1 IFN generation exists in mammals (humans and mice), fish (*D. rerio* and *Oryzias latipes*), insects, (*Drosophila* spp., wasps), molluscs, (oysters), and cnidarians (sea anemone, Hydra, corals) (Table 1) [24]. The cGAS or C6orf150 or male abnormal 21 (Mab21) domain-containing protein 1 recognizes self- and pathogen-derived cytosolic DNA to activate the innate immune response (Figure 1) [30]. Human cGAS (h-cGAS) comprises an unstructured and poorly conserved N terminus (amino acid (AA) residues 1–160) and a highly conserved C terminus (160–513). The C terminus consists of a conserved nucleotidyltransferase (NTase) core domain (160–330) and a Mab21 domain (213–513) that contains a zinc (Zn^2+^)-ribbon structural domain (390–405) [22]. This Zn^2+^ ribbon domain is conserved among all vertebrate cGAS members and cGLRs, excluding three homologs in *Pan paniscus* (bonobo), *Canis familiaris* (dog), and *Taeniopygia guttata* (zebra finch) [22].

Mab21 was first recognized as the fate-determining gene for the morphogenesis of the sensory rays, a male-specific sense organ located in the tail and involved in copulation in the *C. elegans* [31]. The invertebrate *Litopenaeus vannamei* (Whiteleg Shrimp or King Prawn) Mab21 (LvMab21cp) has a similar expression profile to LvSTING and LvIRF and serves as a human analog of the vertebrate cGAS, which recognizes viruses and generates antiviral immune responses [32]. Mouse cGAS (m-cGAS) also contains the Mab21 domain; however, eukaryotic homologs of m-cGAS are more similar to m-cGAS than mouse Mab21-like proteins [22]. Notably, h-cGAS structurally and enzymatically resembles human OAS1, a template-independent nucleotidyl transferase that activates antiviral innate immunity upon recognizing cytosolic short dsRNA (>18 bp) [33,34,35]. Notably, OAS-like proteins also have an evolutionary prokaryotic or bacterial origin through the development of antiviral defense and are independently acquired by eukaryotes [18]. Furthermore, the Zn^2+^-ribbon domain of vertebrate cGAS is absent in OAS1, nematode Mab-21, and mammalian Mab-21-like proteins [22]. The presence of the elongated N-terminal (167 AAs) in vertebrate and cephalochordate (*B. floridae*) cGAS has evolved as an adaptation that is very short in invertebrates (varying from fewer than 7 AAs to 70) [22].

Both h-cGAS and m-cGAS have two DNA binding sites and form a dimeric complex with two dsDNAs [36,37]. Of the five AA residues that are critical for 2′3′-cGAMP binding, only S434 of h-cGAS is non-conserved in mammals, whereas Y436 is conserved in all species. The three remaining residues (K362, R376, and S378) are wholly conserved in cephalochordates and vertebrates but not in arthropods [22]. The details of conserved and non-conserved AA residues in the dsDNA binding sites of cGAS have been discussed elsewhere [22]. The four non-conserved AA residues (R236, K254, K327, and R353) in vertebrates suggest that only double and triple mutations inhibit cGAS’s ability to stimulate type 1 IFN production [22,36]. Thus, the NTase system has evolved as a defense system against foreign and delocalized genetic material that enters the cytosol from the mitochondria and nucleus. These findings demonstrate that GLRs are critical family members of PRRs.

More than three thousand cGLRs with complete active sites representing nearly all major animal phyla have been identified [24]. Furthermore, cGLR sequence analysis has delineated specific evolutionary patterns in animal immunity and protein features that are distinct from bacterial CD-NTase anti-phage defense enzymes [24]. Animal cGLRs form a single family of signaling proteins that share more distantly related homologies with bacterial CD-NTase anti-phage defense enzymes and animal OAS1-like proteins [18,24]. Furthermore, the interaction between STING and CDNs is universally conserved. However, some rare bacteriophages avoid potent innate immune responses by preventing cGAS/STING signaling through major capsid gene mutations in the CBASS [38,39,40]. Even bacteriophages escaping the bacterial cGLR or CBASS generate longer cabRNA that prevents the recognition and activation of CdnEO3.

**STING**-mediated CDN recognition also originated in bacteria as a defense mechanism against bacteriophages. Bacterial STING (b-STING) forms protein filaments to drive oligomerization of Toll/Interleukin 1 receptor (TIR) effector domains and rapid NAD^+^ cleavage [23]. STINGs and bacterial-like STING (bl-STINGs, present in microeukaryotes clustered between bacterial and metazoan sequences) share similarities in their domains [18]. Most new eukaryotic bl-STINGs have four N-terminal alpha helices as seen in human STING (h-STING). Furthermore, bacterial transmembrane domain -STINGs (TM-STINGS) have two N-terminal alpha helices and are more similar to bacterial Toll/Interleukin 1 receptor (TIR)-STING or TIR-STING, indicating eukaryotes and bacteria independently converged on a typical TM-STING domain architecture through domain shuffling [18]. Notably, TIR domains of oyster TIR-STING are related to animal TIRs and differ from the TIR domains of bacterial TIR-STINGs. Eukaryotic TIR-STINGs are rare, supporting the hypothesis that this protein originated from recent animal convergence or convergent domain shuffling [18,41]. Therefore, TM-STING and TIR-STING proteins have evolved due to two independent convergent evolution processes, and bacteria and eukaryotes have used similar proteins by reusing ancient protein domains.

The Drosophila STING (DmSTING or CG1667) is an ortholog to the vertebrate STING. DmSTING has evolutionarily conserved antibacterial (*Listeria monocytogenes*) and antiviral (positive-strand RNA *Drosophila* C virus and other DNA viruses) activity that involves the downstream activation of immune deficiency (IMD) and the Drosophila inhibitory kinase kinase β (dIKKβ) pathway and the subsequent cleavage of Relish (an NF-κB homolog) to generate antimicrobial peptides (AMPs) without the involvement of its cGAS ortholog CG7194. CG7194 lacks both the Zn^2+^-ribbon domain and a positively charged N terminus which are critical for DNA binding [42,43,44,45,46]. Furthermore, DmSTING transferred to mammalian cells induces an NF-κB-dependent immune response. Hence, Drosophila cGLRs, especially cGLR1, sense cytosolic dsRNA to generate the 3′2′-cGAMP that DmSTING recognizes to initiate the downstream innate immune response through activating Relish [47,48].

Drosophila cGLR2 generates 2′3′-cGAMP and 3′2′-cGAMP through an unknown target to generate a protective antimicrobial immune response [48]. The loss of DmSTING in Parkin RBR E3 ubiquitin-protein ligase KO (parkin^−/−^) flies rescues thorax muscle defects, climbing ability, and disrupted mitochondrial morphology of their flight muscles, indicating a critical noncanonical role of DmSTING in cellular and organismal responses to mitochondria stress [49]. Flies lacking parkin and DmSTING exhibit PTEN-induced kinase 1 (PINK1) activation to suppress cell death. Parkin, PINK1, and mitochondrial fidelity play critical roles in the pathogenesis of Parkinson’s disease (PD) in humans [50,51,52]; therefore, it would be interesting to see if a similar h-STING-Parkin-PINK-1 axis exists in human patients with PD. Thus, the cGAS/STING signaling-mediated immune defense mechanism is evolutionarily very ancient, with its origin dating back to prokaryotes/bacteria fighting against invading viruses. Eukaryotes, including humans, have acquired it via convergent domain shuffling and multi-HGT to guard against viruses and self-derived cytosolic DNAs.

## 3. cGAS/STING Signaling Pathway in Cellular and Immune Homeostasis Maintenance

cGAS (a nucleotidyltransferase family member) was initially discovered as an interferon-stimulated gene (ISG) [53]. cGAS is a membrane (inner plasma membrane leaflet)-bound cytosolic protein/enzyme that quickly recognizes invading DNA viruses or the DNA released from an adjacent dying cell, such as cancer cells and DNA contained in the extracellular vesicles (EVs) entering the cell via endocytosis (Figure 1) [54,55,56]. However, membrane-bound cGAS frequently and strongly recognizes pathogen-derived DNA more than host-derived cytosolic dsDNA [55]. Thus, membrane binding of the cGAS is a strategy to avoid its overactivation against cytosolic dsDNA to prevent the increased incidence of autoinflammatory or autoimmune diseases. For example, membrane-bound cGAS is more present in phagocytic cells such as macrophages, dendritic cells (DCs), and neutrophils (which have extremely low levels of cGAS and STING) than in non-phagocytic cells [30,54,57]. However, neutrophils express SRY-box transcription factor 2 (SOX-2) in their cytoplasm to recognize cytosolic microbial DNA in a sequence-specific manner to induce a pro-inflammatory immune response [58]. Endocytosis-mediated foreign DNA uptake promotes spleen tyrosine kinase (SYK) and cGAS recruitment to the endosome [56]. However, mutant cGAS (with a defective N-terminal binding site supporting cGAS interaction with the plasma membrane) swims in the cytosol and recognizes host-derived cytosolic dsDNA (mitochondrial and nuclear DNA released in the cytosol due to genotoxic nuclear and mitochondrial damage) more easily than invading pathogen-derived DNA (Figure 1) [30,54,55,59]. cGAS recognizes micronuclei through chromothripsis-mediated cGAS concentration, activating STING-mediated type 1 IFNs and NF-κB-dependent cytokines and chemokine release (Figure 1) [60,61,62].

cGAS located in the nucleus does not recognize and cleave nuclear DNA as it is not naked and bound to histone proteins to form nucleosomes, comprising chromatin fibers to form chromosomes [63,64,65]. Under normal conditions, nuclear cGAS extensively interacts with the acidic patch of the histone H2A–H2B heterodimer through its second DNA binding site and nucleosomal DNA, which locks cGAS into a monomeric stage that hinders the cGAS enzymatic activity by burying the cGAS DNA binding site B and blocking cGAS dimerization, which is critical for cleaving genomic DNA (Figure 1) [66,67,68,69]. The mutation inhibiting cGAS–acidic patch interaction abolishes the inhibitory action of nucleosomes against cGAS enzymatic activity and increases genomic DNA recognition and cleavage. Furthermore, nuclear cGAS is tightly tethered to intact nuclear chromatin by a salt-resistant interaction that does not involve domains required for cGAS enzymatic activity in generating cGAMP [63]. The cGAS tethering surface is evolutionarily conserved, and a change in single AA massively results in cGAS-mediated cleavage of nuclear DNA. Notably, nuclear cGAS is critical for recognizing the viral DNA of viruses replicating in the nucleus [70]. For example, herpes simplex virus-1 (HSV-1) infection induces cGAS release from the chromatin into the nuclear soluble fraction that senses viral DNA to synthesize cGAMP and mRNA expression for type 1 IFNs and other ISGs. Thus, nuclear cGAS limits HSV-1 replication and infection. Along with viral infections, cGAS/STING signaling is critical for generating a protective immune response against bacterial, parasitic, and fungal infections to maintain immune homeostasis [40,71].

Cytosolic cGAS recognizes cytosolic dsDNA depending on the base pair (bp) length independent of the sequence [27,72]. A dsDNA length of 36 bp is ideal for cGAS enzymatic activity in generating cyclic dinucleotides (CDNs) called 2′-3′ (most common), 3′-2′ (*Drosophila melanogaster*), and 3′3′ (bacterial) cGAMP that activate a downstream adaptor protein called the stimulator of interferon genes (STING) or endoplasmic reticulum IFN stimulator (ERIS) [72]. cGAS-induced CDN is the only known mammalian CDN that chemically differs from bacterial CDNs by having one noncanonical 2′-5′ phosphodiester bond, combining guanine (G) and adenine (A), and one canonical 3′-5′ phosphodiester bond, joining A and G [73,74,75,76]. Therefore, the cGAS product is sometimes known as 2′3′cGAMP to differentiate it from the bacterial CDN called 3′3′cGAMP [77]. It is important to note that cGAMP can also be transported through gap junctions and other transporters (SLC19A1or reduced folate carrier 1 (RFC1) and SLC46A2; cGAMP importers in human macrophages) to adjacent cells to activate their STING to promote the antiviral immunity independently of type 1 IFN signaling involvement [78,79,80,81]. Thus, cGAMP serves as an immunotransmitter for adjacent immune cells.

Notably, all STING homologs have a conserved central c-di-GMP-binding domain (CBD) and dimerization domain (DD) [22]. For example, human STING (hSTING) has four N-terminal transmembrane domains (TMs), a central CBD with a DD that protrudes in the cytosol from the ER, and a C-terminal tail (CTT is critical for transducing downstream signals) [82,83]. CBD + CTT, called the CTD (carboxy-terminal domain), binds to the bacterial CDNs and 2′3′-cGAMP produced in mammalian cells to initiate the downstream signaling responsible for generating type 1 IFNs, pro-inflammatory cytokines and chemokines, and autophagy [75,84,85,86]. The CTT domain is only present in vertebrate STING, except its homologs, which are present in amphibians such as *Xenopus tropicalis* and *Xenopus laevis* [22]. Notably, the STING CTT domain is a critical determinant of downstream signaling, such as the activation of type 1 IFNs and NF-κB-dependent pro-inflammatory response intensity [87]. For example, the difference in the CTT sequence of human and zebrafish STING is responsible for increased NF-κB activation upon STING activation in zebrafish and increased type 1 IFN generation as opposed to NF-κB-dependent pro-inflammatory molecule generation in humans [87]. However, the most primitively diverged vertebrate lineage (*Callorhinchus milii*, ghost shark) has a human-like CTT and therefore does not show NF-κB overactivation upon STING activation [87].

cGAS activation may lead to the STING-dependent canonical signaling pathway inducing type 1 IFN release, NF-κB activation-mediated release of different cytokines (TNF-α, IL-1β, IL-6, and IL-12), and chemokines (CXCL1, CXCL9, and CCL5), MAP kinase, and STAT6-pathway-dependent upregulation of co-stimulatory molecules (CD40 and CD86) (Figure 1) [88,89,90,91]. For example, constitutive type 1 IFNs produced in low quantities in the absence of acute infections through tonic signaling and their increased production during microbial infections and other inflammatory conditions are critical to maintaining immune homeostasis [92,93,94,95,96]. cGAS-dependent noncanonical signaling may involve different STING-dependent events, such as cellular senescence (involving STING-dependent production and release of the senescent-associated secretory phenotype or SASP), autophagy (independent of TANK-binding kinase 1 or TBK1 activation and type 1 IFN generation), cellular apoptosis (T cell death), and cellular senescent and fibrosis through a newly identified axis called cGAS/STING/PKR-like endoplasmic reticulum kinase (PERK)/eukaryotic translation initiation factor 2alpha (elF2α), which induces a global mRNA translation arrest and operates independently of the classical ER stress response [97,98,99,100,101,102,103,104]. Apoptosis and autophagy are critical to maintaining immune homeostasis during disease development and in sterile and infectious inflammatory conditions [105,106,107].

Interestingly, RNAs do not directly bind and activate cGAS but promote cGAS condensate formation comprising cGAS and dsDNA to enhance the cGAS activity even in the presence of low levels of cytosolic dsDNA [108]. Thus, cGAS can recognize low levels of cytosolic dsDNA in the presence of cytosolic RNA to generate an optimum pro-inflammatory immune response to maintain cellular homeostasis. However, in the abundance of cytosolic dsDNA, RNA competes with it in phase-separated granules of cGAS to inhibit its activity [56,108]. It is crucial to note that cGAS activation is responsible for producing more type 1 IFNs than any other cytosolic PRR (e.g., TLR7 and TLR9) recognizing cytosolic dsDNA. We have described the details of cGAS/STING signaling and its regulation elsewhere [30,56,109,110].

**During cellular homeostasis**, inactive or apo-STING exists as a bilayer with head-to-head and side-by-side packing via its ligand binding domain (LBD); this assembly holds two endoplasmic reticulum (ER) membranes together [111,112]. This STING-ER assembly prevents the STING ER from exiting the ER and inhibits TBK1 recruitment to support the autoinhibited STING stage. Furthermore, the inactive STING interacts with a calcium (Ca^2+^) sensor called stromal interaction molecule 1 (STIM-1) that retains its position in the ER membranes and, upon activation (recognition of 2′-3′ cGAMP), moves to the Golgi complex, where it activates or phosphorylates TBK1 (Figure 1) [113,114,115,116]. The active STING appears as a curved polymer that could deform the ER membrane for its exit and anterograde transportation [111]. For example, the filamentous structure of the STING/2′,3′-cGAMP complex adopts a bent monolayer assembly mediated by the LBD and transmembrane domain (TMD) [111,117].

TBK1 alone is dispensable for the STING-induced NF-κB-mediated immune response in murine and human immune cells [118]. Furthermore, TBK1 is redundant with IKKε to induce NF-κB upon STING activation but is critically needed for IRF3 activation and type 1 IFN release [118]. The maintenance of cellular redox homeostasis is also critical to keep a check on the STING activity. For example, glutathione peroxidase 4 (GPX4), maintaining cellular redox homeostasis, is critical for STING activation [119]. GPX4 deficiency increases cellular lipid peroxidation (LPO) and inhibits the cGAS/STING activity induced by STING carbonylation at C88, inhibiting its trafficking from the endoplasmic reticulum (ER) to the Golgi complex [119]. Therefore, the ER retention/retrieval sequence RIR is critical to retaining the STING or ERIS on the ER membrane and its integrity. Furthermore, ER stress during infectious, inflammatory, and autoimmune diseases increases cytosolic Ca^2+^ levels, which disrupts STING-STIM-1 bonding, allowing STING to move from the ER to the ER–Golgi intermediate complex (ERGIC) and Golgi complex [120,121,122,123]. STING ER exit protein (STEEP), or chromosome X open reading frame 56 (CXORF56), is critical for the STING ER exit as it mediates increased phosphatidylinositol-3-phosphate (PIP3) production, ER membrane curvature, and COPII-dependent STING ER-to-Golgi trafficking in response to dsDNA-dependent STING activation [124].

STEEP deficiency is associated with a defective cGAS/STING signaling pathway. Therefore, STING translocation from the ER to ERGIC and Golgi apparatus (GA) is critical in the cGAS/STING signaling-dependent canonical and noncanonical signaling pathways. The details of STING trafficking are mentioned elsewhere [125]. Notably, STING overactivation is prevented by its quick degradation in the lysosome, as upon its arrival to the Golgi complex, which is critical for TBK1 activation and downstream signaling, activated STING is quickly phosphorylated by serine/threonine UNC-51-like kinase (ULK1/ATG1) that degrades in the Rab7-positive endolysosomes [126,127]. CDNs generated due to the cGAS activity induce ULK1 dissociation from its repressor called adenosine monophosphate (AMP)-activated protein kinase (AMPK) [127]. The free ULK1 phosphorylates Golgi STING to prepare for its degradation in the lysosome. However, activated AMPK promotes cGAS/STING signaling independently of ULK1 in murine macrophages and embryonic fibroblasts [128].

The endolysosomal degradation of ubiquitinated STING in murine macrophages requires endosomal complexes required for the transport (ESCRT) pathway involved in microautophagy for its recognition [129,130,131,132]. ESCRT deficiency or inhibition is associated with an overactivated cGAS/STING signaling pathway. Furthermore, the constant tug-of-war between Toll-interacting protein (TOLLIP, which directly interacts with STING at the ER to stabilize it) and IRE1α-lysosome (STING degrader) is critical for maintaining cell and tissue immune homeostasis [133]. TOLLIP deficiency is associated with reduced resting-state STING protein levels and severely reduced cGAS/STING signaling to maintain cellular and immune homeostasis. The ER-associated degradation (ERAD, a cellular process of recruiting and retro-translocating misfolded ER proteins for ubiquitin–proteasomal degradation in the cytosol) protein complex, called the suppressor of lin-12-like (SEL1L)–HMG-CoA reductase degradation 1 (HRD1), increases STING levels under homeostasis in macrophages to escape from STING-driven inflammation [134,135].

The activated STING phosphorylates TBK1, which moves to the nucleus and activates different transcription factors, such as IFN-releasing factor 3 (IRF3) and NF-κB, to synthesize and release type 1 IFNs and NF-κB-dependent pro-inflammatory cytokines and chemokines, which is critical for antitumor and antiviral immune responses (Figure 1) [136]. However, the STING translocation to the ERGIC acts as a membrane source for microtubule-associated protein 1 light chain 3 (MAP1LC3/LC3) lipidation, forming an autophagosome in the WD repeat domain, phosphoinositide interacting 2 (WIPI2), and autophagy related-5 (ATG5) independently of ULK and the VPS34/BECLIN kinase complex [100]. STING-mediated autophagy involves its direct interaction with WIPI2, which recruits WIPI2 to STING-positive vesicles for LC3 lipidation and autophagosome formation [137,138]. During this process, STING and phosphatidylinositol-3-phosphate (PtdIns3P) competitively bind to the FRRG motif of WIPI2 for mutual inhibition between STING-induced and PtdIns3P-dependent autophagy [138,139]. The STING-WIPI2 interaction is critical to removing cytosolic DNA through autophagy and attenuating cGAS/STING signaling-dependent type 1 IFN release and other NF-κB-dependent pro-inflammatory cytokine and chemokine releases [137,138]. Thus, this pathway induces autophagy among infected cells and does not induce type 1 IFNs and NF-κB-dependent cytokine/chemokine generation and serves as a primitive host defense mechanism in sea anemone, *N. vectensis*, upon stimulation with cGAMP [100].

*N. vectensis* cGAS synthesizes 3′3′-cGAMP, which is recognized by their STING in nucleobase-specific contacts, which is absent in human STING (hSTING) [140]. Notably, sea anemone STING binds to 2′, 3′ cGAMP indistinguishably from hSTING, trapping a unique structural conformation not induced by 3′,3′ CDNs. However, with the increase in animal phyla such as Mollusca, for example, the Pacific oyster (*Crassostrea gigas* or *C. gigas*), the involvement of cGAS/STING signaling-mediated production of primitive interferon-like protein (IFN-LP) has been observed upon viral infection as a defense mechanism [141,142,143]. For example, the Cg-cGAS/cg-STING of *C. gigas* recognizes polyinosinic acid/polycytidylic acid (poly I:C), a synthetic analog of dsRNA and dsDNA, to produce C*g*-IFN-LP in a Cg-TBK1- and IRF (Cg-IRF1/8)-dependent manner [143]. CgSTING also recognizes bacterial CDNs to provide an antimicrobial defense by producing cg-IFN-LP- and NF-κB-dependent pro-inflammatory cytokines in a TBK1/IKKε-dependent manner [144,145].

Furthermore, the IFN-LP released from *C. gigas* induces apoptosis and phagocytosis of hemocytes in vivo and kills cancer cells in vitro [146]. *C. gigas* also expresses an IκB-related kinase ε (IKKε) called *CgIKKε-like* that critically regulates NF-κB by phosphorylating the inhibitor of NF-κB (IκB) called CgIκBs and in the regulation of IFN gene expression by phosphorylating IFN regulatory factors (IRFs) such as CgIRF8 and CgIRF1 [147,148]. CgIκB4 is critical for the NF-κB-dependent immune response against LPS, peptidoglycan (PGN), and poly I:C [149]. Interestingly, CgIKKε-like transfection into human cells induces NF-κB and ISRE activation. Hence, human 2′3′ cGAMP achieves universal signaling by exploiting a deeply conserved STING conformational intermediate to activate TBK1-mediated IRF3-dependent type 1 IFNs and NF-κB-mediated synthesis and the release of pro-inflammatory mediators [140]. Therefore, cGAS/STING signaling is a primitive innate immune defense mechanism that, in lower animals (invertebrates) such as sea anemones, provides a host defense without inducing type 1 IFN production, which has evolved with the hierarchy of animal phyla, especially in chordates. However, IFN-like molecules’ production has emerged as an antiviral defense mechanism in Mollusca phylum. Thus, cGAS/STING signaling, through several mechanisms (autophagy, apoptosis) and producing different regulatory molecules, such as type 1 IFNs and NF-κB-dependent cytokines, regulates cellular and immune homeostasis.

## 4. Altered cGAS/STING Signaling Dysregulates Immune Homeostasis and Makes Us Sick, like Other Known PRRs

The deficiency or aberrant activation of previously well-studied PRRs (TLRs, NLRs, ALRs, CLRs, and RLRs) is associated with increased susceptibility to different infections, cancers, immunodeficiency diseases, and autoinflammatory and autoimmune diseases, as discussed elsewhere [1,5,6,7,12,13,88,150,151,152,153,154]. Like these different PRRs, the dysregulation of a critical cGLR in humans called cGAS disturbs immune homeostasis and leads to different diseases. For example, an overactive cGAS/STING signaling pathway is associated with different autoinflammatory and autoimmune diseases, such as ataxia–telangiectasia (AT), Aicardi–Goutières syndrome (AGS), and STING-associated vasculopathy with onset in infancy (SAVI), erosive inflammatory arthritis (EIA), and several other inflammatory diseases, as discussed in detail elsewhere [79,110,155]. Furthermore, an aberrantly activated cGAS/STING signaling pathway in the endothelial cells of diabetic retinopathy (DR) patients and animal models has been observed [156]. STING overexpression in the endothelial cells of DR patients induces senescence, inflammatory changes, and capillary degeneration at early stages, causing early progression. Notably, STING knockout (KO) and STINGGT (loss-of-function mutation) mice were protected from the early onset of DR symptoms due to a decrease in TBK1, IRF3, and NF-κB phosphorylation, critical mediators of cGAS/STING signaling-dependent type 1 IFNs, other pro-inflammatory molecules (cytokines and chemokines), and senescence generation [156].

On the other hand, decreased cGAS/STING signaling increases susceptibility to various viral and bacterial infections due to lowered innate and adaptive immune responses. Decreased cGAS/STING signaling increases the severity of these infections as several pathogens have evolved different mechanisms to evade this system along with other PRRs [157,158,159]. In addition, bacteriophages infecting bacteria where the cGAS/STING system evolved also develop mechanisms to evade their CBASS to establish infection [38]. However, during fungal infections such as *Candida albicans*, STING translocation to the phagosome decreases the antifungal immune response [160]. STING translocated to the phagosome binds to the Src kinase via its N-terminal 18 AA motif to inhibit Syk kinase activity to suppress the release of antifungal pro-inflammatory cytokines and chemokines. STING-deficient macrophages exert a better antifungal immune response than WT macrophages and prevent infection dissemination [160]. Therefore, the cGAS/STING system is critical for infectious diseases and their progression to life-threatening infections.

Chronic low-grade inflammation is one of several factors predisposing humans to developing cancers, and chronic low-grade type 1 IFN generation through the activation of cGAS/STING signaling promotes tumorigenesis and its further spread and proliferation, as discussed in detail elsewhere [56,161]. Furthermore, cGAS/STING signaling dysregulation in cancer patients’ myeloid immune cells (MICs) increases the cancer’s severity [109]. Additionally, cellular STING restricts aerobic glycolysis independently of its role in innate immunity by targeting hexokinase 2 (HK2) activity [162]. This anti-HK2 activity of STING restricts tumor aerobic glycolysis and promotes antitumor immunity. For example, in colorectal cancer (CRC) cells, an increased lactate level decreases STING activity and associated antitumor immunity. Furthermore, STING serves as a cell-intrinsic metabolic checkpoint and restricts aerobic glycolysis to promote the antitumor immune response [162]. Thus, cGAS/STING signaling regulates tumorigenesis and cancer cell proliferation and metastasis depending on the cancer stage and type. This indicates that cGAS/STING signaling is critical for host defense and maintenance of immune homeostasis; pathogens and cancer cells have evolved different strategies to evade this [39,163].

Furthermore, chronic inflammation is a critical player in neurodegenerative diseases such as Alzheimer’s disease (AD), Parkinson’s disease (PD), amyotrophic lateral sclerosis (ALS), and multiple sclerosis (MS), as discussed in detail elsewhere [164,165,166]. Besides several pro-inflammatory mechanisms governed by different PRRs, cGAS/STING signaling is also critical for neuroinflammation and its progression to neurodegeneration [167,168,169,170]. Human microglia and astrocytes (critical players in neuroinflammation and neurodegeneration) also express these (cGAS and STING) proteins of the cGLR family [171]. The loss of ataxia–telangiectasia-mutated (ATM) protein causes a pleiotropic disease called ataxia–telangiectasia (AT), and its patients also develop neurodegeneration, specifically cerebellar Purkinje neurodegeneration, along with cancer and other immune dysfunctions. The neurodegeneration seen in AT patients occurs due to overactive STING in their microglia in response to increased cytosolic dsDNA due to failure of the DNA repair mechanism [172,173].

Additionally, aging increases the cytosolic dsDNA released from perturbed mitochondria in old or aging microglia, which induces cGAS/STING signaling activation, triggering reactive microglial transcriptional states, neurodegeneration, and cognitive decline [174]. Thus, the cGAS/STING signaling pathway is a significant driver of inflammaging, or age-related inflammation, in the peripheral organs and the brain that can be targeted to combat the neurodegeneration and cognitive decline associated with aging and seen in older people. It is noteworthy that human neurons do not express STING. Therefore, dsDNA accumulation in neurons cannot induce cGAS/STING-dependent inflammatory self-neuronal damage [173,175]. Furthermore, the human/mammalian cGLR (cGAS)/STING system is critical for normal development (embryonic hematopoiesis and brain development) [176,177]. Therefore, the cGLR system is a critical mediator of the inflammatory phenotype once dysregulated under diverse pro-inflammatory conditions including infections, cancers, inflammaging, and neurodegeneration. For example, conditional STING KO mice (STING is knocked out in nestin (primarily expressed in neuron- expressing cells) develop an autism-like phenotype [176]. Hence, cGAS/STING signaling regulates different immune mechanisms to maintain immune homeostasis, and its altered activity predisposes the host to different infections and diseases, such as autoimmunity, aging, and cancers.

## 5. cGLRs (cGAS/STING Signaling) Cross-Talk with Other PRRs to Maintain Immune Homeostasis and Regulate Inflammation

### 5.1. TLR and cGAS/STING Signaling Interaction

TLRs are critical PRRs with a broad ligand (PAMPs and DAMPs) range, as mentioned elsewhere [5,6,7]. Therefore, exploring TLRs’ impact on cGLRs (cGAS/STING signaling) and vice versa would be interesting. For example, during *Staphylococcus aureus* skin infection, TLR and cGAS/STING signaling pathway activation regulates the expression of approximately 95% of genes in macrophages within the first four hours of infection [178]. Furthermore, TLR signaling, by promoting IL-1β production and neutrophil recruitment, helps clear the pathogen (*S. aureus*). In contrast, the absence of STING signaling is critical for this; however, STING activation is critical for type 1 IFN production early in the infection. Interestingly, heat-killed *S. aureus* stimulates type 1 IFN production through TLR signaling more strongly than live *S. aureus* induces this through STING activation [178]. This immunological discrepancy occurs due to the hypoxia induction in infected macrophages by live *S. aureus* that stimulates the expression of most TLR/STING-independent genes that may impact cGAS/STING signaling. For example, type 1 IFNs produced during *S. pyogenes* infection suppress IL-1β-mediated inflammation [179]. Furthermore, viral infections (*Influenza A*) suppress antibacterial immunity by preventing IL-1β production by suppressing NF-κB activation [180].

**Figure 2:**
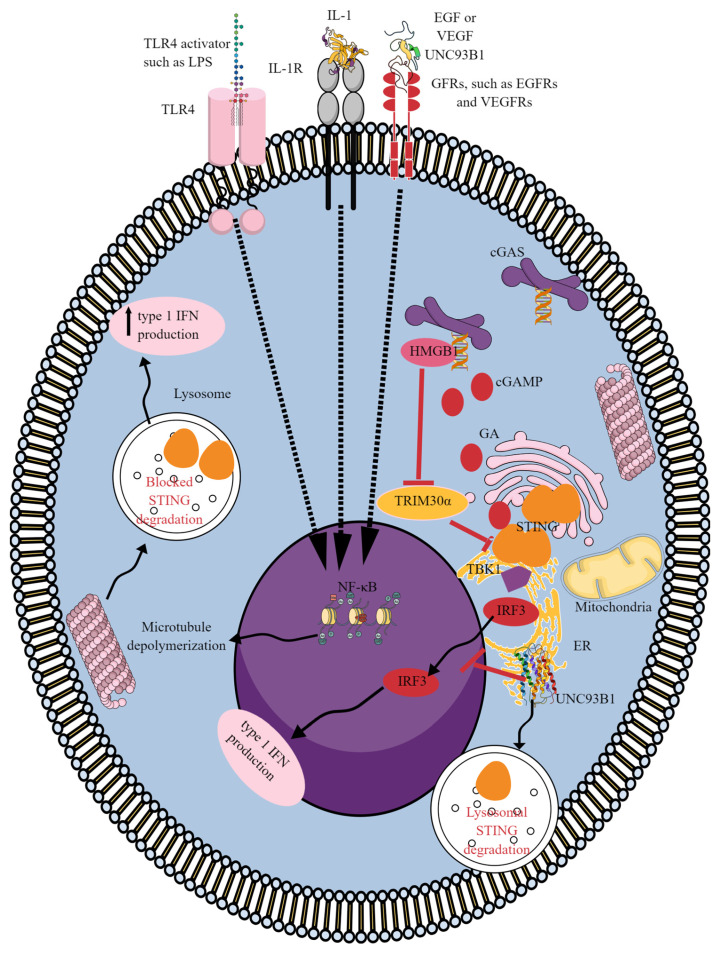
TLRs and other NF-κB activators regulate cGLR (cGAS/STING signaling) activation. TLR4, IL-1R, and GFRs (EGFRs and VEGFRs), by activating NF-κB, induce microtubule depolymerization that blocks STING degradation in the lysosome. This further stimulates type 1 IFN production by increasing the activity of activated STING to produce type 1 IFNs. HMGB1 bound to the DNA blocks the activity of TRIM30α, a negative STING regulator that further enhances STING-dependent type 1 IFN generation. UNC93B1, a TLR signaling regulator, also inhibits cGAS/STING signaling by different mechanisms, such as inhibiting IRF3’s translocation to the nucleus and promoting lysosomal STING degradation. Details are mentioned in the text.

TLR4 activation and other activators of NF-κB, such as IL-1 receptor (IL-1R), TNF-receptors (TNF-Rs), and growth factor receptors (GFRs; epidermal growth factor, or EGF; and vascular endothelial growth factor, or VEGF) enhance STING-dependent type 1 IFN production by promoting microtubule depolymerization to inhibit lysosomal STING degradation (Figure 2) [181]. This increases the time and level of activated STING for the robust type 1 IFN production for a potent antiviral host defense. Additionally, several gain-of-function mutations of STING also inhibit its lysosomal trafficking and degradation, causing ligand-independent STING activation and subsequently autoinflammatory diseases [181]. Thus, TLR-mediated cGAS/STING signaling regulation depends on the type of TLR activated.

For example, TLR9 (localized to the ER membrane of resting immune cells such as macrophages and DCs, including plasmacytoid DCs or pDCs) shuttles to the endosome to initiate the NF-κB-dependent cytokine and type 1 IFN-dependent pro-inflammatory immune response against pathogen-derived unmethylated deoxycytidylyl-deoxyguanosine dinucleotide (CpG) DNA trapped in endosomes, lysosomes, and endolysosomes [182,183]. The trafficking of TLR9 from the ER to the GA involves Unc-93 Homolog B1 (UNC93B1, TLR signaling regulator), which further regulates TLR9 loading to COPII^+^ vesicles originating from the ER [184,185]. COPII^+^ vesicles transport TLR9 to the plasma membrane [186]. UNC93B1 deficiency is associated with complete loss of intracellular TLRs (TLR3 (recognizes 40–50 bp dsRNA, and the strength of binding and signaling increases with the RNA length), TLR7, TLR8, and TLR9) in immune cells, such as splenic macrophages and DCs [187,188]. On the other hand, UNC93B1 negatively regulates the cGAS/STING signaling pathway by blocking IRF3 nuclear translocation, reducing the stability of STING by facilitating autophagy–lysosome degradation that can be reversed by lysosome inhibitors, suppressing *IFN-β* promoter activity and the transcriptions of *IFN-β*, *ISG54*, and *ISG56* genes (Figure 2) [189]. Therefore, exploring the effect of UNC93B1 loss on the cGAS/STING signaling pathway would be interesting because it is associated with the functional loss of intracellular TLRs, which regulate or affect the cGAS/STING signaling pathway in immune cells.

Additionally, cytosolic high-mobility group box 1 protein (HMGB1) is a low-specificity DNA-binding protein containing two HMG boxes for DNA binding and a C-terminal acidic tail that resides in an approximately 60 AA long conserved intrinsically disordered region (IDR) [190,191]. The binding of HMGB1 to the cytosolic self-DNA aggravates the TLR9-mediated pro-inflammatory immune signaling pathway due to increased recognition of cytosolic DNA that may enhance STING-dependent type 1 IFN release to clear pathogenic infections or exaggerate sterile pro-inflammatory diseases such as autoinflammation and autoimmunity [30,181]. Deleting the HMGB1 gene in mouse embryonic fibroblasts impairs TLR3, TLR7, and TLR9 activation and associated IRF-3 and NF-κB activation in the presence of their cognate ligands (nucleic acids) [192].

Immunogenic nucleic acid binds more strongly to HMGB1 than less immunogenic nucleic acid. Furthermore, HMGB2 binds to B-DNA but not RNA, and HMGB3 binds to both DNA and RNA to activate endosomal TLRs [192]. Thus, TLR9 activation has the potential to upregulate cGAS/STING signaling by activating NF-κB and IRF3 as well. Furthermore, HMGB1 can directly activate cGAS/STING signaling by binding to long U-turn DNA in the cytosol [193]. HMGB1 release during senescence also suppresses E3 ligase tripartite motif protein 30α (TRIM30α, a negative regulator of STING) by binding to its promoter site, which stabilizes STING to induce STAT6 activation in response to genotoxic stress for p21 (cyclin-dependent kinase inhibitor 1A) activation in cancer cells (Figure 2) [194]. Thus, HMGB1 alone or activating different TLRs (TLR4, TLR9, TLR3, and TLR7) can activate cGAS/STING signaling to control the inflammatory immune response [195]. Further studies are needed to explore the direct interaction between TLR and cGAS/STING signaling to understand the immune homeostasis and pathogenesis of inflammatory and infectious diseases.

For example, frameshift variant mutations replacing the acidic tail of HMGB1 with an arginine-rich base tail, causing nucleolar dysfunction due to its altered subnuclear localization, have been discovered to be associated with a rare pro-inflammatory condition called brachyphalangy, polydactyly, and tibial aplasia syndrome (BPTAS), a rare complex malformation syndrome, requiring an exploration of cGAS/STING signaling along with other PRRs [191]. HMGB1 lacking the entire IDR (Del IDR) or the sequence after the frameshift position (Del FS) does not reside in the nucleolus. It is determined by the arginine residue within the sequence created by the frameshift mutation. The hydrophobic patch contributes to nucleolar arrest [191]. Therefore, the accumulation of the HMGB1 frameshift mutant in the cell dysregulates nucleolar function and is responsible for the cytotoxicity. Hence, it is critical to study the impact of the HMGB1 frameshift mutant on the TLRs, recognizing it as a DAMP, and the impact on DNA binding capacity, altering the TLR9 and cGAS/STING signaling pathways, to understand the developmental, immunological, and inflammatory defects observed in BPTAS patients. Therefore, TLRs, through different PAMPs and DAMPs, are critical regulators of cGLRs such as cGAS/STING functioning and inflammatory and infectious diseases.

### 5.2. ALR and NLR Interaction with cGAS/STING Signaling

**Figure 3:**
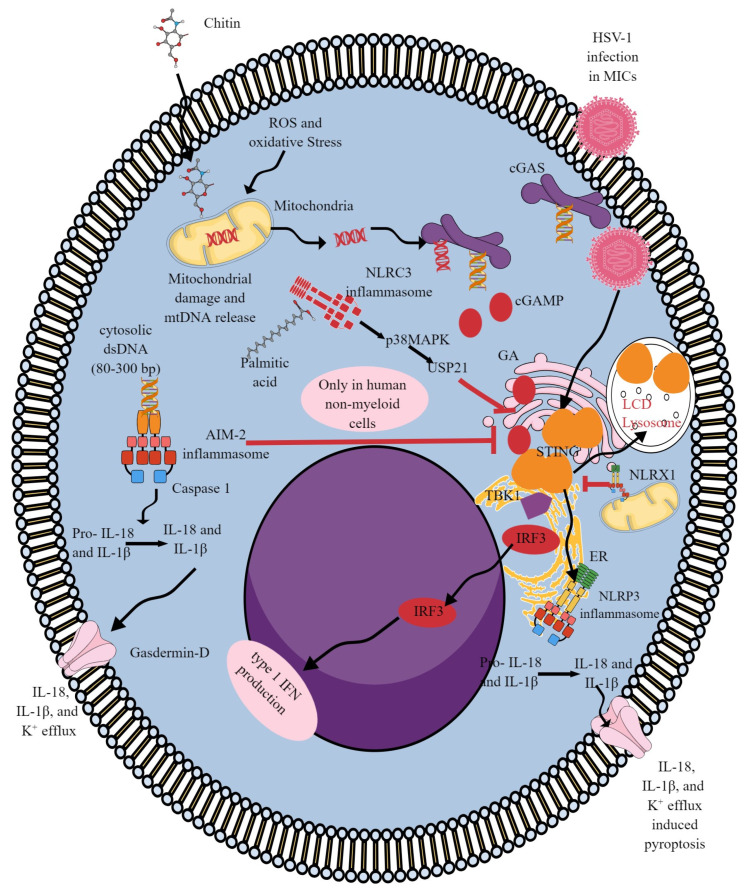
The interaction between ALR, NLR, and cGLR (cGAS/STING signaling pathway). AIM-2 (an ALR) activation in response to the cytosolic DNA in non-myeloid cells inhibits cGAS/STING signaling activation by causing an increased K^+^ efflux that is dispensable in human myeloid cells. AIM-2 activation induces caspase 1 activation to induce the generation of the pro-inflammatory cytokines IL-1β and IL-18 and gasdermin-D channel formation for K^+^ efflux. In human myeloid cells, cGAS/STING activation in response to the cytosolic dsDNA induces K^+^ efflux upstream of NLRP3 activation, which involves STING trafficking to the lysosome and LCD. cGAS/STING/NLRP3 signaling axis activation been observed during HSV-1 infection, where STING-dependent type 1 IFNs are not produced, and instead NLRP3-dependent IL-1β, IL-18, and gasdermin formation occurs, causing pyroptosis. Chitin-derived polysaccharide adjuvants also activate the cGAS/STING/NLRP3 signaling axis by increasing mitochondrial ROS generation. NLRC3 activation in response to palmitic acid blocks cGAS//STING signaling by activating p38MAP and USP21. NLRX1 residing on the outer mitochondrial membrane also inhibits cGAS/STING signaling by directly interacting with STING and blocking TBK1 activation and the downstream type 1 IFN signaling pathway. Details are mentioned in the text.

AIM2 activation in the presence of sizeable cytosolic self- or viral dsDNA (80–300 bp) is associated with the inactivation of the cGAS/STING signaling pathway due to increased potassium (K^+^) efflux and blockage of STING and TBK1 interaction by the activated AIM2 inflammasome, as discussed in detail elsewhere (Figure 3) [30,196]. However, AIM2-mediated inflammasome activation in response to cytosolic dsDNA is dispensable in human myeloid cells. Instead, cGAS/STING signaling induces a cell death program by initiating K^+^ efflux upstream of the nucleotide oligomerization domain-like receptor protein 3 (NLRP3) inflammasome (Figure 3) [197]. This process involves STING trafficking to the lysosome, triggering membrane permeabilization to induce lysosomal cell death (LCD). During HSV-1 infection, STING promotes NLRP3 localization to the ER, facilitating its deubiquitination and activation to form an inflammasome to release pro-inflammatory cytokines (Figure 3) [198]. STING’s interaction with NLRP3 attenuates K48- and K63-linked polyubiquitination of NLRP3 to activate the inflammasome. Furthermore, manganese (Mn^2+^)-induced neurotoxicity also involves cGAS/STING/NLRP3 axis-induced inflammation and induces Tau pathology due to an overactivated cGAS/STING signaling pathway that further activates the NLRP3 inflammasome [199]. Thus, the cGAS/STING/NLRP3 pathway comprises the default inflammasome response during microbial infections in human myeloid immune cells (MICs), which can be exploited to target pathology in inflammatory diseases (Figure 3).

Functionally different mutations have converged to form the cGAS/STING/NLRP3 axis in myelodysplastic syndrome (MDS, a group of cancers in which blood cells are poorly formed and do not work typically) to direct ISG production, pyroptosis, and myeloid lineage skewing [200]. The cytosolic escape of mitochondrial DNA (mt-DNA) also activates the cGAS/STING/NLRP3 axis to induce pyroptosis of nucleus pulposus (NP) cells to aggravate inflammation during intervertebral disc (IV) degeneration (Figure 3) [201]. Oxidative stress, by regulating the mitochondrial permeability transition pore (mPTP) for the cytosolic escape of mitochondrial DNA (mtDNA), plays a critical role in activating cGAS/STING/NLRP3 axis-dependent NP cell pyroptosis during IV degeneration, causing lower back pain (LCB) [201]. Another study has shown that chitin-derived polysaccharide adjuvants with a high degree of deacetylation also upregulate the cGAS/STING/NLRP3 axis via increasing mitochondrial reactive oxygen species (ROS) generation (Figure 3) [202]. Hence, chitin-derived polysaccharide adjuvants activate the cGAS/STING/NLPR3 axis to enhance antigen-specific Th1 responses to increase the vaccine’s antigenicity. Furthermore, increased STING, AIM2, IRF3, NLRP3, Caspase-1, and IL-1β levels in the serum of diabetic nephropathy (DN) patients and their whole-blood immune cells have shown overexpressed STING and AIM2 mRNAs [203]. This indicates the involvement of STING-mediated NLRP3 inflammasome activation and pyroptosis in DN patients. Therefore, the cGAS/STING/NLPR3 axis can be targeted in sterile inflammatory conditions such as MDS, DN, and infections. Future studies are required in this direction as NLRP3 dysfunction serves as a significant driver of MDS, and cGAS/STING signaling in human myeloid cells works upstream of NLRP3 via STING-mediated LCD and initiates K^+^ efflux-associated pyroptosis (Figure 3) [197,204,205]. cGAS inhibition reversed the myeloid lineage bias in MDS in mice. Furthermore, hydrogen sulfide (H_2_S, an anti-inflammatory molecule) also inhibits the cGAS/STING/NLRP3 axis to protect against choline-induced cardiotoxicity [206,207].

NLR family CARD-containing 3 (NLRC3) protein leucine-rich repeats (LRRs) at the C terminal recognize viral dsDNA (HSV-1) and dsRNA [9]. The recognition of the cytosolic dsDNA by NLRC3 enhances its ATPase activity, unleashing its interaction with STING and TBK1, leaving them free to recognize cytosolic cGAMP and induce type 1 IFN production [208]. Thus, during cellular homeostasis, the nucleotide-binding domain (NBD) of NLRC3 interacts with STING and TBK1, which prevents proper STING trafficking from the ER to peri-nuclearpuncta region (critical for STING activation) [209,210,211]. Thus, NLRC3 helps in maintaining cellular homeostasis by keeping a check on cGAS/STING signaling-dependent type 1 IFN, TNF-α, and IL-6 synthesis and release to prevent autoinflammation and autoimmunity in response to the low levels of cytosolic dsDNA. However, NLRC3 activation inhibits NLRP3 (NALP3 or cryopyrin) inflammasome by competing for apoptosis-associated speck-like protein containing a CARD (ASC) adaptor protein for pro-caspase-1 binding [212]. Thus, NLRC3 activation under diverse inflammatory and infectious conditions may activate cGAS/STING signaling for releasing type 1 IFNs but simultaneously block the cGAS/STING/NLRP3 axis in MICs, including macrophages.

Furthermore, NLRC3 activation in T cells and dendritic cells (DCs) also negatively regulates T cell activation through different mechanisms, including STING- and TBK1-mediated ones, to prevent autoimmunity and exaggerated inflammation [208,213,214,215,216]. For example, NLRC3 activation in DCs attenuates their antigen presentation function through p38 MAPK activation, which also phosphorylates USP21 (ubiquitin-specific peptidase 21, a nuclear/cytoplasmic shuttling deubiquitinase) at Ser538 to inhibit STING activity by hydrolyzing its K27/63-linked polyubiquitin chain, which limits their ability to activate CD4^+^T cells and their polarization to pro-inflammatory Th1 and Th17 cells to prevent against autoinflammation and autoimmunity (Figure 3) [216,217,218]. Hence, NLRC3 activation affects myeloid and lymphoid (T cells) cells’ immune function to control exaggerated inflammation. For example, the MICs of people with obesity are desensitized to STING activation as saturated fatty acids inhibit STING activation by activating NLRC3 [219]. Furthermore, people with obesity having head and neck cancer are unresponsive to STING-stimulation-based antitumor approaches due to saturated fatty acid (palmitic acid)-mediated NLRC3 activation in their MICs, blocking STING activation and mediated type 1 IFN release (Figure 3) and the associated T-cell-mediated antitumor immune response [219,220]. We need further studies to understand NLRC3 and cGAS/STING or cGLR interaction in different diseases.

NLRX1 (a reside on the outer mitochondrial membrane) negatively regulates cGAS/STING signaling by binding to STING, which decreases its interaction with TBK1 to generate a type 1 IFN-dependent immune response during human immunodeficiency virus-1 (HIV-1) infection along with other DNA virus infections, such as HSV-1 and vaccinia virus (Figure 3) [221]. NLRX1 was the first NLR discovered to inhibit type 1 IFN signaling [222,223,224].

NLRP4, another NLR protein, degrades TBK1 upon activation to alleviate the cGAS/STING-dependent immune response by recruiting the E3 ubiquitin ligase DTX4 at its NACHT domain for Lys48 (K48)-linked polyubiquitination at Lys670 of TBK1 [225]. TBK1 degradation blocks IRF3 phosphorylation and its translocation to the nucleus to activate different ISGs and type 1 IFNs. Therefore, this interdependency in these two PRR systems (cGLRs and NLRs) further strengthens their evolutionary divergence and need to escape from pathogens, which causes inflammation and inflammatory diseases upon dysregulation [30]. For example, in terms of their evolutionary aspects, inflammasomes such as ALRs and NLRs are the youngest (evolved in vertebrates) of both TLRs and cGAS, which might have evolved to take care of their overactivation to prevent exaggerated inflammation (autoinflammation and autoimmunity) [30,226,227]. Therefore, along with the NLRP3 inflammasome that forms the cGAS/STING/NLRP3 axis in different inflammatory conditions, NLRs negatively regulating the cGLR (cGAS/STING pathway) system have also evolved to protect the host, including humans, from autoinflammatory and autoimmune diseases. It will also be critical to investigate the role of these negative regulatory NLRs in different cancers where cGAS/STING signaling is dysregulated to contribute to tumor growth and metastasis.

### 5.3. RLRs Impact cGLRs or cGAS/STING Signaling

RIG-1, melanoma differentiation-associated protein 5, or MDA5, and LGP2 are cytosolic PRRs, which recognize viral genetic materials such as ssRNA, dsRNA, and dsDNA, but no known examples of RLR-mediated recognition of ssDNA are available [13]. For example, RIG-1 recognizes relatively short dsRNAs (22–500 bp) and ssRNAs, whereas MDA5 recognizes larger dsRNAs (500–1000 bp) depending on their length and degree of complementarity [188]. Activated RLRs (RIG-1 and MDA5), after recognizing their corresponding ligands, interact through their CARD domains with the CARD domains (CARD-CARD interaction) of mitochondrial antiviral-signaling protein (MAVS), the major adaptor protein of the signaling pathway [228]. MAVS anchored in the mitochondria, the peroxisome, and mitochondrial-associated membranes (MAMs) activates TBK1 and IKKε to further activate IRF3, IRF7, and NF-κB-dependent type 1 IFNs and pro-inflammatory cytokines, as discussed in detail elsewhere [13,14,228,229,230].

NLRP12, expressed mainly in DCs and neutrophils, dampens the RLR-mediated immune response by interacting with the tripartite motif protein 25 (TRIM25), which blocks its potential to ubiquitylate and activate RIG-1 during RNA virus infection [231]. NLRP12 is critical for the cytosolic sensor for heme plus PAMP-mediated PANoptosis, inflammation, and pathology induced by the formation of PANoptosome (a complex comprising NLRP12, caspase 12 (CASP12), ASC, and RIPK3 with and without NLRP3) [232]. Thus, NLRP12 is anti-inflammatory only during RNA virus infections activating RIG-1. Furthermore, stress granules, or SGs (molecular condensates that form in response to various stresses such as viral dsRNA), are also critical for the controlled activation of RLRs. For example, in the absence of the SG nucleators Ras-GTPase-activating protein-binding protein 1/2 (G3BP1/2) and ubiquitin Associated Protein 2 Like (UBAP2L), overactivation of RLRs occurs in the presence of dsRNAs (viral and host-derived), causing excessive immune-mediated apoptosis [233]. Thus, SGs are cellular shock absorbers, which converge to maintain cellular homeostasis by dampening toxic/overactive immune responses and viral replication.

Downstream RLR (RIG-1 and MDA5 via MAVS) signaling involves TBK1/IKKε activation-induced type 1 IFN production, which raises the question of whether RLR signaling activation would impact cGLR (cGAS/STING) signaling involving TBK/IKKε activation-mediated type 1 IFN production or tilt cGAS/STING signaling to work independently of type 1 IFN production, e.g., via inducing autophagy, apoptosis, and senescence [56,109]. For example, overactivated RLR signaling in response to increased ROS production due to dysfunctional mitochondria accumulation dysregulates autophagy [234,235]. MAVS activation downstream of RLR activation during influenza A virus (IAV) infection through its M2 protein increases ROS production and decreases autophagy [236]. Hence, MAVS is critical for maintaining mitochondrial homeostasis via autophagy [237]. Furthermore, increased ROS production also oxidizes cysteine 147 of mouse STING, which is equivalent to cysteine 148 of human STING, and suppresses type 1 IFN production [238]. For example, oxidized STING is defective in its polymerization. It loses its function, which is also critical for its noncanonical autophagy function in an ATG5-dependent manner without involving other autophagy regulators, including Beclin1, Atg9a, ULK1, and p62 [101]. However, RIG-1 activation supports autophagy during viral infection by involving MAVS-tumor necrosis factor receptor-associated factor 6 (TRAF6)-Beclin-1 axis independently of ROS production, which is not involved in STING-dependent autophagy [239]. Thus, ROS-induced RLR activation indirectly controls cGAS/STING function and decreases autophagy under diverse conditions. Autophagy dysregulation is critical as it maintains immune cells’ renewal, differentiation, and homeostasis, as discussed in detail elsewhere [105,240,241]. Further studies are required in this direction.

Furthermore, cytosolic RNAs colocalize with phase-separated condensates of cGAS and dsDNA to promote cGAS-containing phase separation and increase cGAS activity in the presence of low cytosolic dsDNA condensate formation [108]. Therefore, it would be interesting to explore how RLR activation in the presence of cytosolic RNA and DNA would impact cGAS/STING activity, or vice versa. Once the cytosolic dsDNA increases, RNA competes with it in phase-separated granules of cGAS to inhibit its activity [56,108]. Also, STING has been identified as an RLR signaling cofactor and essential signaling adaptor protein which directs an innate immune response against DNA viruses, which can overlap with the host innate immune response during DNA virus infection or even in conditions where host-derived cytosolic dsDNA accumulation occurs [14,114,242]. Hence, it is critical to understand RLR and cGLR (cGAS/STING signaling) interaction during different infections and inflammatory diseases, such as autoinflammation and cancers.

## 6. Future Perspectives and Conclusions

PRRs are significant drivers of innate immunity associated with the adaptive immune response and have a role in embryonic development. For example, TLRs were first recognized as Toll proteins critical for dorsoventral body patterning during *D. melanogaster* embryonic development, and later studies showed that they also played a role in clearing pathogens, such as bacteria and fungi, by controlling the secretion of antimicrobial peptides (AMPs) such as drosomycin [243,244,245]. Another study in the laboratory of Charles A. Janeway Junior identified the existence of its homolog in humans, known as TLR4, which recognizes the Gram-negative bacterial PAMP called lipopolysaccharide (LPS) or endotoxin to initiate the protective pro-inflammatory innate immune response to clear the pathogen at the expense of bystander local inflammatory tissue damage that can become worse if the infection is uncontrolled or severe such as in cases of sepsis [246]. Thus, TLRs’ discovery filled the long-existing gap of pathogen recognition by immune cells as TLR1-10 in humans and TLR1-13 in mice recognize a wide range of PAMPs, such as LPS, proteins (flagellin), lipoteichoic acid (LTA), DNA, and RNA. Later studies proved that TLRs also recognize a wide range of DAMPs and PAMPs/MAMPs to regulate the immune response and maintain homeostasis [4,5,7]. Similarly, NLRs also play a critical role in recognizing cytosolic pathogens and DAMPs, which can also be exploited for infectious and inflammatory diseases, including cancers and autoimmunity [247].

IFNs were first described as antiviral factors or proteins released by cells in response to viral infections in the 1950s [248,249]. However, the molecular mechanism behind their (type 1 IFNs) release from all nucleated cells was only understood after the discovery of STING in the early 2000s (2008 and 2009) [113,114,242,250]. Thus, the discovery of the cGAS/STING signaling pathway of cGLRs has also solved the mystery of IFN release during viral infections, specifically DNA viruses and the transfer of antiviral immunity to adjacent cells before the virus can infect and kill them [26,27,251,252]. For example, along with type 1 IFNs released to provide antiviral signaling to adjacent cells, the infected and dying cells will also release cGAMP, which will be taken by adjacent cells through different transporter proteins to develop an antiviral immune response to protect themselves. Furthermore, alum has been used as an adjuvant for over 100 years.

The discovery of cGAS/STING signaling has also solved the mystery of the mechanism of action of alum as an adjuvant. For example, alum works through activating the cGAS/STING signaling system in APCs such as DCs and macrophages by inducing the release of DNA from dying cells at the injection site along with activating NLRP3 inflammasomes to release IL-1β and IL-18 to induce inflammation and increases Ag uptake and sustains Ag presentation [253,254,255,256,257]. However, another study indicated that alum-mediated NLRP3 activation and -dependent IL-1β release are dispensable for its adjuvant effect [258]. Therefore, alum-induced cGAS/STING activation is critical for its high adjuvanticity. Hence, the discovery of cGAS/STING signaling, a part of the cGLR system, solved the mystery of type 1 IFN release and mediated antiviral stage development in nearby uninfected cells and alum’s adjuvant effect. Furthermore, manganese (Mn^2+^ also critically regulates cGAS/STING signaling by increasing cGAS sensitivity to dsDNA) salts exert an adjuvant effect by activating the cGAS/STING signaling pathway in APCs such as DCs [259,260]. Therefore, the discovery of the cGAS/STING system has permitted the development of a class of adjuvants and innate immune system regulators to develop vaccines and immunomodulators for infectious diseases and cancers [56,109,261,262]. Additionally, these PRRs do not work in isolation but interact with each other directly and indirectly through unexplored mechanisms, which need to be studied to understand their role in maintaining immune homeostasis and preventing autoinflammation, autoimmunity, infections, and different cancers.

Hence, PRRs have welcomed cGLRs (cGAS/STING signaling pathway) as their family member. In contrast, other PRRs not only support them directly or activate NF-κB but also stop them from preventing exaggerated inflammation for safety. Thus, the PRR family is an excellent example of the saying that united we stand, divided we fall; they work together to maintain homeostasis, including immune homeostasis and host safety and longevity. Failure of this controlled PRR family regulation becomes dangerous for the host. It increases their susceptibility to different immunodeficiency disorders, autoinflammation, autoimmunity, many cancers, and even increased aging.

## Figures and Tables

**Table 1:** Different cGLRs (including mammalian cGAS), their ligands, cleaved products (cyclic dinucleotides, and CDNs such as 2′3′-cGAMP and/or 3′3′-cGAMP) activating STING and downstream signaling molecules throughout the animal kingdom from metazoans to mammals. Kindly see the article text and referenced articles for details.

Species	cGLR	Ligand/Product	STING	IKKε	TBK1	NF-κB	IRF3	IRF7
*Homo sapiens*/Human	cGAS	dsDNA/2′3′-cGAMP	+	+	+	+	+	+
*Mus musculus*/Laboratory mouse	cGAS	dsDNA/2′3′-cGAMP	+	+	+	+	+	+
*Gallus gallus*/Red junglefowl	cGAS	dsDNA/2′3′-cGAMP	+	+	+	+	-	+
*Xenopus tropicalis*/Western clawed frog	cGAS	dsDNA/2′3′-cGAMP	+ (without CTT domain)	+	+	+	+	+
*Branchiostoma floridae*/Florida lancelet	2 cGAS homologs	dsDNA/2′3′-cGAMP	+, 2 STING candidates (STING-1 and STING-2,	+	+	+	-	-
*Danio rerio*/Zebrafish	2 cGAS	Cytosolic dsDNA/2′3′-cGAMP	+	+	+	+	+	+
*Strongylocentrotus purpuratus*/Pacific purple sea urchin	?	?	-	+	+	+	-	-
*Ceanorhabidits elegans*/Roundworm	-	-	-	+	-	-	-	-
*Drosophila melanogaster*/Common fruit fly	*Dm*-cGLR1 and *Dm*-cGLR2	dsRNA/3′2′-cGAMP and 2′3′-cGAMP	+	-	+	+	-	-
*Tribolium castaneium*/Red flour beetle	*Tc*-cGLR	dsRNA/2′3′-cGAMP	+	-	+	+	-	-
*Microplitis demolitor*/Wasp	*Md*-cGLR	dsRNA/2′3′-cGAMP	+	-	+	+	-	-
*Frankliniella occidentalis*/Western flower thrip	*Fo*-cGLR	dsRNA/2′3′-cGAMP	+	?	+	+	-	-
*Nicrophorus vespilloides*/Common sexton beetle	*Nves*-cGLR	dsRNA/2′3′-cGAMP	+	?	+	+	-	-
*Aethina tumida*/Small hive beetle	*At*-cGLR	dsRNA/2′3′-cGAMP	+	?	+	+	-	-
*Asbolus verrucosus*/Blue death feigning beetle	*Av*-cGLR	dsRNA/2′3′-cGAMP	+	?	+	+	-	-
*Trichogramma pretiosum*/Wasp	*Tp*-cGLR	Unknown/2′3′-cGAMP	+	-	+	+	-	-
*Chlamydophila felis*/Cat flea	*Cf*-cGLR	Unknown/2′3′-cGAMP	?	?	+	+	?	?
*Pocilloporidae damicornis*/Cauliflower coral	*Pd*-cGLR	Unknown/2′3′-cGAMP	+	-	+	+	-	?
*Crassostrea gigas*/Pacific oyster	*Cg*-cGLR1	dsDNA/2′3′-cUA	+	+	+	+	-, but have cgIRF1	-, but have cgIRF8
*Crassostrea virginica*/Eastern oyster	*Cv*-cGLR1	dsDNA/2′3′-cUA	+	+	+	+	-, but have cgIRF1	-, but have cgIRF8
	*Cv*-cGLR2	Unknown/Unknown						
	*Sp*-cGLR1	dsRNA/3′3′-cUA						
*Stylophora pistillata*/Stony coral	*Sp*-cGLR2	Unknown/2′3′-cGAMP	+	-	?	+	?	?
	*Sp*-cGLR3	dsRNA/3′3′-cAA						
*Amphimedon queenslandica*/Sponge	?	?	-	-	+	+	-	-
*Exaiptasia pallida*/Glass anemone	*Ep*-cGLR	Unknown/2′3′-cGAMP	+	-	+	+	-	-
*Monosiga brevicollis*/Choanoflagellate	*Mb-cGAS*	dsDNA/3′3′-cGAMP	+	-	-	-	-	-
*Nematostella vectensis*/Starlet Sea anemone	Nv-cGAS or nvA7SFB5.1	dsDNA/3′3′-cGAMP	+	-	+	+	-	-
*Hydra magnipapillata*/Hydra vulgaris	*Hv*-cGLR	dsRNA/2′3′-cGAMP	+, 3 STING candidates (STING-1, STING-2, and STING-3)	+	-	+	-	-

+ = present; - = absent; ? = not known/unsure; cUA, cyclic UMP-AMP; cAA, cyclic diAMP.
